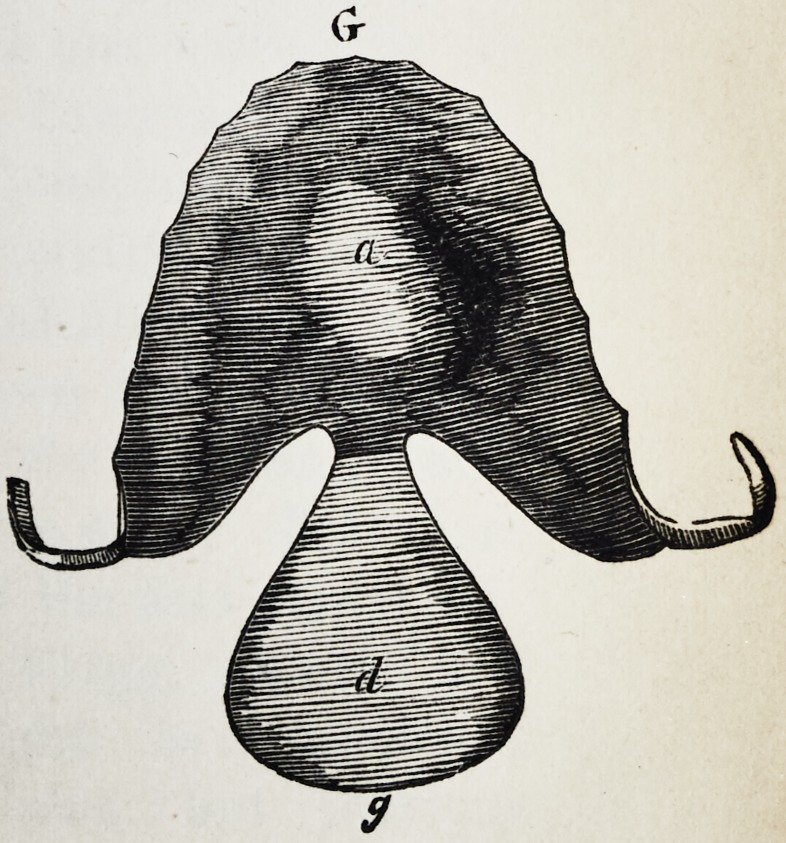# A New System of Artificial Palates

**Published:** 1867-12

**Authors:** Wilhelm Suersen

**Affiliations:** Berlin, Prussia.


AMERICAN JOURNAL

OF

DENTAL SCIENCE.

Vol. I. THIRD SERIES--DECEMBER, 1867. No. 8.

### ORIGINAL COMMUNICATIONS.

### ARTICLE I.

### *A New System of Artificial Palates.*

By DR. WILHELM SUERSEN, SR., Berlin, Prussia.

(Provisional Extracts trom a Lecture " On the restoration of a distinct utterance by means of a new system of artificial palates to be employed in cases of congenital and acquired defects of the palatine organs." Held at Hamburg on the 5th of August, 1867, at the 6th Annual Meeting of the Central Association of German Dentists. A short sketch extracted from the report on the sitting of the above-named meeting.)

THE theory of the formation of sounds, which forms the basis of my system, may be set forth in a few simple propositions. These propositions are based on the works of Merkel and Brucke, especially on that of the last-named author.*

*" Anatomie und Physiologie des menschlichen Stimm- und Sprachorgans von Dr. Carl Ludwig Merkel." Leipzig, 1863, und "Grundzuge der Physiologie der Lautbildung von Professor Dr. Ernst Brucke." Wien, 1856.

The complete stenographic report, containing the exact description of the technical manipulations, is now printing, and will first be published in the organ of the above-mentioned association: "German Quarterly Journal for Dentology."

1. The air, having passed through the larynx and communicated verberations to the chordæ vocales, (which produces the sound,) finds at this point two ways open to it: the one leading below the velum palati through the cavity of the mouth; the other above the velum palati through the cavity of the nose.

2. The nasal sound arises: whenever the air current finds no impediment to its entering the cavity of the nose, and in this respect it is quite indifferent, whether the air can freely issue from here through the nostrils, or whether, while the nostrils are closed, it is made to reverberate only within the cavity of the nose. In proof of the first proposition Brucke (l. c.) states a simple experiment: "Hold a feebly burning light, (say a wax-taper) before the face, in such a manner, that the flame may be met by the breath issuing from the nose, but not by that from the mouth. Then pronounce a pure vowel (*a* for instance) without a nasal sound, and the flame will remain entirely unmoved. But it will at once begin to flutter, when you pronounce the same vowel with a nasal sound."

3. All letters, with the sole exception of *m* and *n*, (to wit in German,) may be pronounced both with and without a nasal sound.

4. The two letters *m* and *n*, on the other hand, can be pronounced only through the nose; in pronouncing the former letter, we close the gates of the lips, in pronouncing the latter, we shut the tongue-gate.

5. In order to be able to pronounce all letters distinctly, it is accordingly, necessary, (besides other conditions, which are far away from our present subject) to separate the cavity of the mouth from the cavity of the nose by means of muscular motion.

6. That separation is, under normal conditions, effected: on the one hand, by the velum palati, which strains itself (consequently by the levator and tensor palati;) but on the other hand, also by a muscle, which, to my knowledge has in connection with these operations not yet received a sufficient amount of attention—I mean the *constrictor pharyngeus superior.* This muscle contracts itself, during the utterance of every letter pronounced without a nasal sound, just as the levator palati does. The constrictor muscle contracts the cavum pharyngo-palatinum, the pharynx-wall bulging out—and it is chiefly on the action of this muscle that I base the system of my artificial palates.

7. These palates, which in all their parts are made of hard caoutchouc, consist of a teeth plate suitably attached to existing teeth and which, at the same time, covers the fissure in the hard palate (if such a fissure exists). Where the fissure commences in the velum, that plate terminates in an apophysis broad enough for filling up the defect. This apophysis is at the same time of such thickness as to keep up a contact between the high edges forming the sides of the apophysis and the two halves of the velum even when the levator palati is in activity. To bring about this contact the more surely, the high edges forming the sides do not rise straight, but obliquely towards the outside. The lower surface of the apophysis, turned towards the mouth lies on about an equal level with the velum, *if the latter is raised by the levator palati.* But when the velum hangs losely downwards, the back part of the artificial palate is lying over it. This back part, accordingly, fills up the cavum pharyngo-palatinum, and in such a manner as not to impede the entrance of the air into the cavity of the nose when the constrictor pharyngous superior is inactive. Thus the patients can without any impediment breathe through the nose. But as soon as the constrictor contracts the cavum pharyngo-palati (this happens, as I will repeat for the sake of clearness, in the utterance of every letter, with the exception of *m* and *n*) the muscle already named reclines against the vertical back-surfaces of the obturator. By this operation the air-current is prevented from entering the cavity of the nose and compelled to take its way through the mouth and thus the utterance loses its nasal sound. To the existence of those vertical surfaces, and consequently to the thickness of that part of my palates, which fills up the fissure in the soft palate and the cavum pharyngo-palatinum I must attach special importance. But for that thickness, the levator palati, when it rises upwards, would not remain in contact with the side-edges of the obturator, nor would the constrictor pharyngeus be able to effect a sufficient termination if the portion of the obturator nearest to it consisted only of a thin plate.''

---

The lecturer now presents three patients. First, Mr. W., joiner, 21 years old, afflicted with a constitutional fissure of the hard and of the soft palate. The patient reads some sentences from a book, *without* the artificial palate, after which he reads the same sentences *with* it, and the difference in the pronunciation is so considerable as to call forth the general plaudits and loud shouts of approbation of the assemblage.

The same marks of approbation are repeated, when the second patient of the name of N., 45 years old, laboring under a considerable acquired defect of the velum, has read, first *without* and then *with* the artificial palate.

Still more enthusiastic, if possible are the acclamations when the third patient, Mrs. A., about 30 years of age and also suffering from an acquired defect of the soft palate, is presented to the auditors. This patient is without the apparatus hardly intelligible, even to those sitting nearest to her whereas with the help of it her utterance is absolutely normal and regular.

These three patients declare in answer to the questions addressed to them, that the apparatus does not cause them the slightest inconvenience, nay that, on the contrary, they would feel very uncomfortable without it, and

Mrs. A., moreover informs the auditors, that formerly the effort she was obliged to make in speaking, always used to cause her pain in the chest; but that those sufferings have entirely disappeared, since she has been using the artificial palate.

---

In conclusion the lecturer alludes to the artificial palates invented by Dr. Kingsley and which are known to have for some years past created considerable sensation in the odontological literature. Those palates, it will be remembered, consist of a soft and moveable piece of caoutchouc which (in cases of congenital defects of the velum) is supported by the side-halves of the velum palati and follows all its movements. Then the lecturer proceeds: "A comparison of the Kingsley-palates with mine proves in favor of the latter:

1. My palates are so firm, that even in the event of their being unskilfully handled by the patients, they cannot get into disorder. They are much more simple than the Kingsley-apparatus, and all the other constructions which had previously been proposed.

2. The Kingsley-palates in consequence of their being based on the action of the levator palati, can be used only in cases of a constitutional palate-fissure, when the side-halves of the velum palati exist. *My* palates, on the other hand, are applicable in *all* cases, for there will hardly ever have been an instance, in which even the pharynx has been destitute of its muscles. Lastly.

3. The Kingsley-palates require renewal after a short use and frequently already after a few months, because the elastically vulcanized caoutchouc soon gets soft in the mouth and produces a disagreeable smell, whereas *my* palates *never* require such renewal.

---

By a unanimous resolution of the meeting the principal prize, the great golden medal, was awarded to the lecturer.

---

*Explanation of the Illustrations to the Lecture of Dr. Wilhelm Suersen, Sr.*

Illustration I. Case of an acquired defect of the soft palate.

Figure A. Representation of the mouth without the apparatus.
" B. The apparatus in situ.
" C. Side view of the apparatus.
" D. The apparatus seen from the back.
" E. The apparatus seen in front.
" F. The apparatus seen from below.
" G. The apparatus seen from above.

The plate (a) and its narrow and thin apophysis (i) which extends from the boundary (b) of the hard palate to the commencement of the defect (c) serve only as supporters to the real thick obturator (d.)

The latter lies in the pharyngo-palatine hollow so that the lower surface of the obturator turned towards the mouth, is about on the same level, as the rest of the velum palati (e.) Against the vertical side (f) and back-edges (g) of the obturator the walls of the pharynx lean, if the latter is contracted by a contraction of the superior constrictor of the pharynx. But if the muscle just mentioned is not in activity, the obturator does not touch the pharynx-wall. The contraction of the constrictor superior, therefore, closes the valve, formed with the help of the obturator, between the cavity of the mouth and that of the nasal bone, while any relaxation of the above-mentioned muscle immediately re-opens that valve.

Illustration II. Case of a constitutional fissure of the hard and soft palate.

### Plate I.

### Plate II.

Fig. A. to G. as in Illustration I. The designation of the letters likewise is the same as there.

The thickness of the obturator begins where the fissure in the soft palate commences. With the high side-edges (h) of the fore-part of the thick obturator (which edges ascend, not straight, but obliquely towards the outside) the side-halves of the fissured velum palati (e) are in constant contact, even the latter is raised by the action of the muscular levator palati. The proportions of the back part, which in the same manner as in the case of an acquired defect, fills up the cavum pharyngo-palati are as in Illustration I.

K. (See Fig. A. and B.) the two halves of the fissured uvula.

## Figures and Tables

**A f1:**
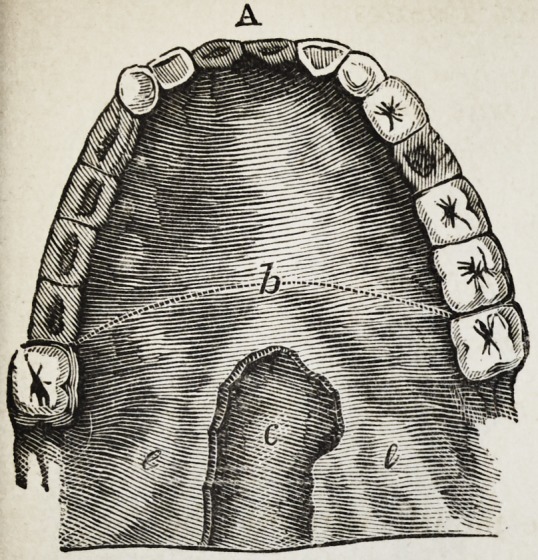


**B f2:**
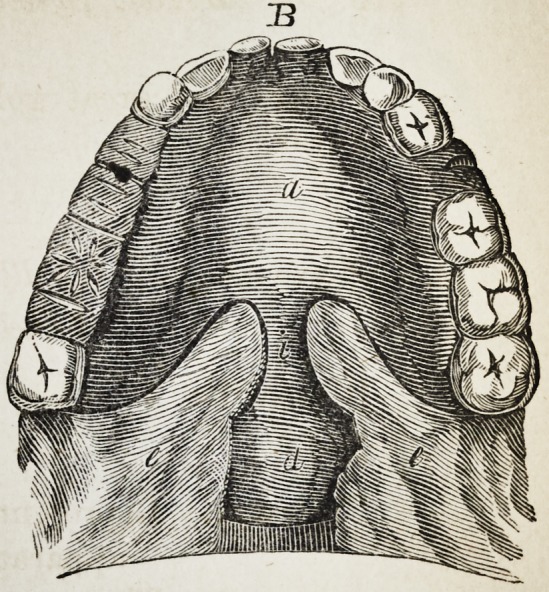


**D f3:**
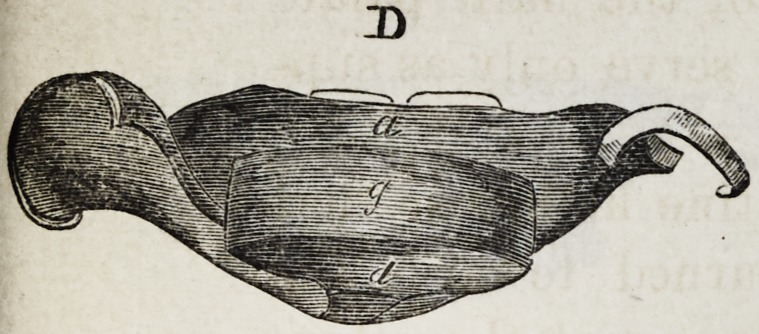


**C f4:**
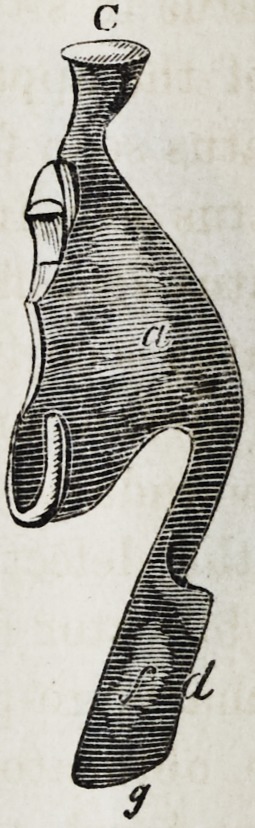


**E f5:**
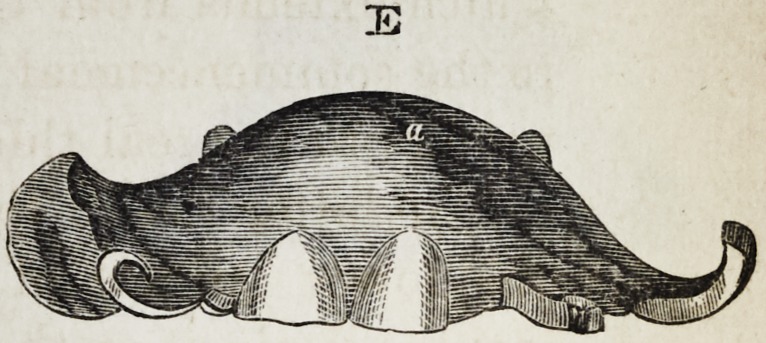


**F f6:**
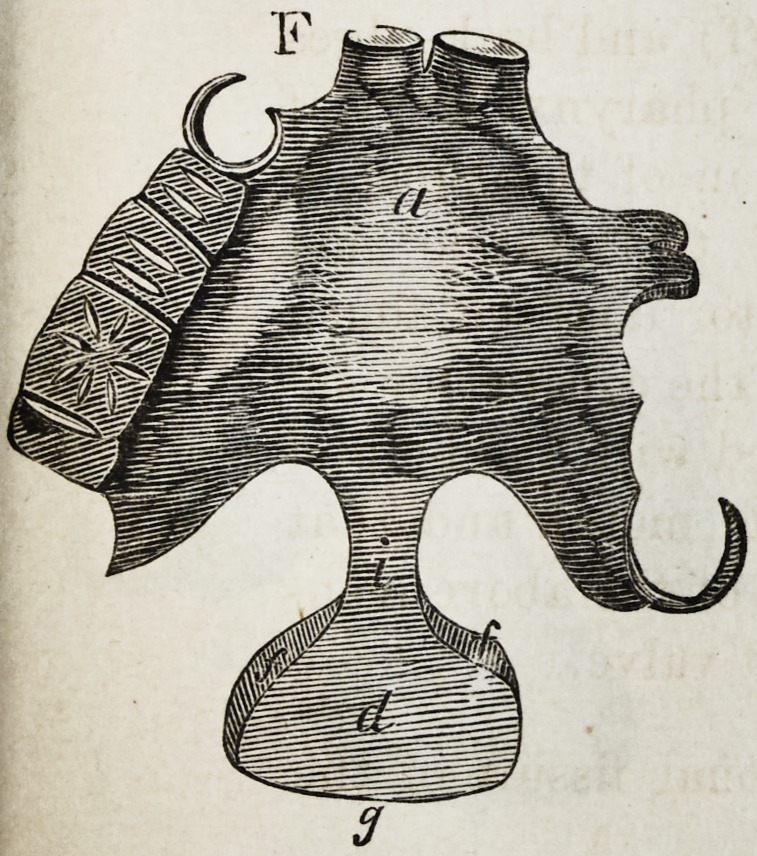


**G f7:**
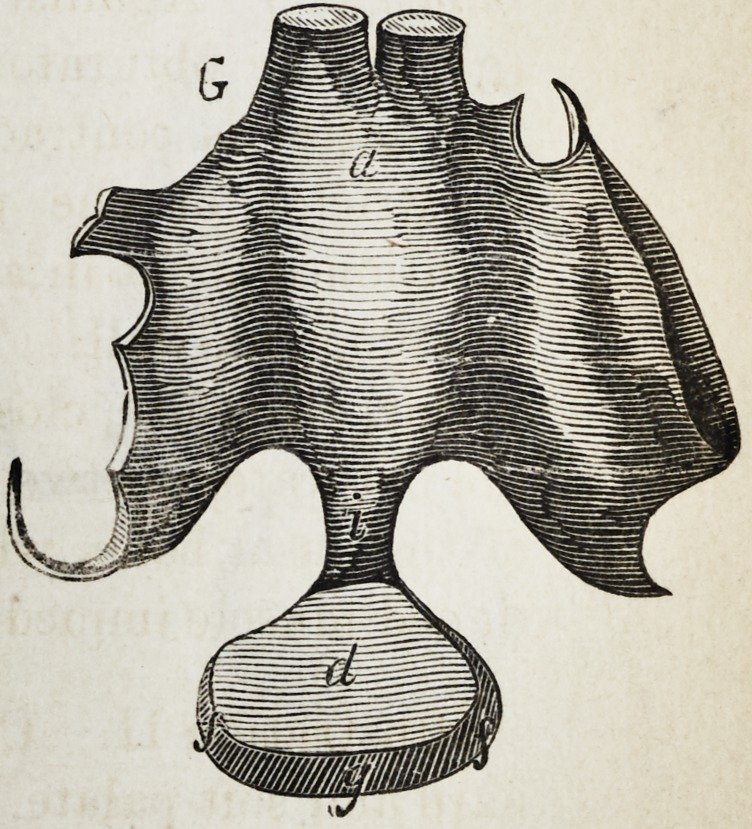


**A f8:**
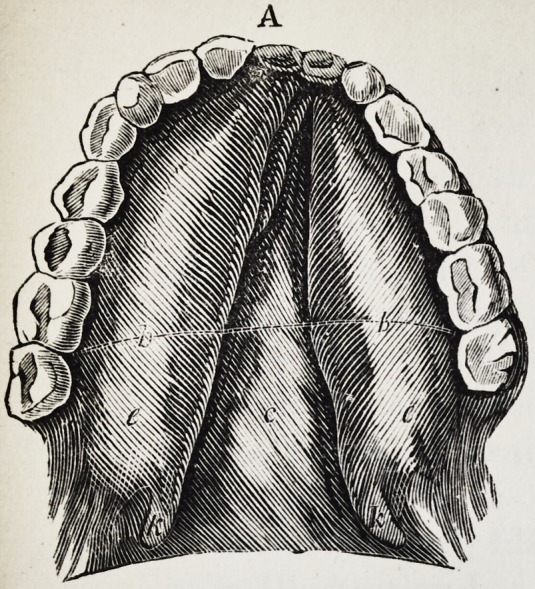


**B f9:**
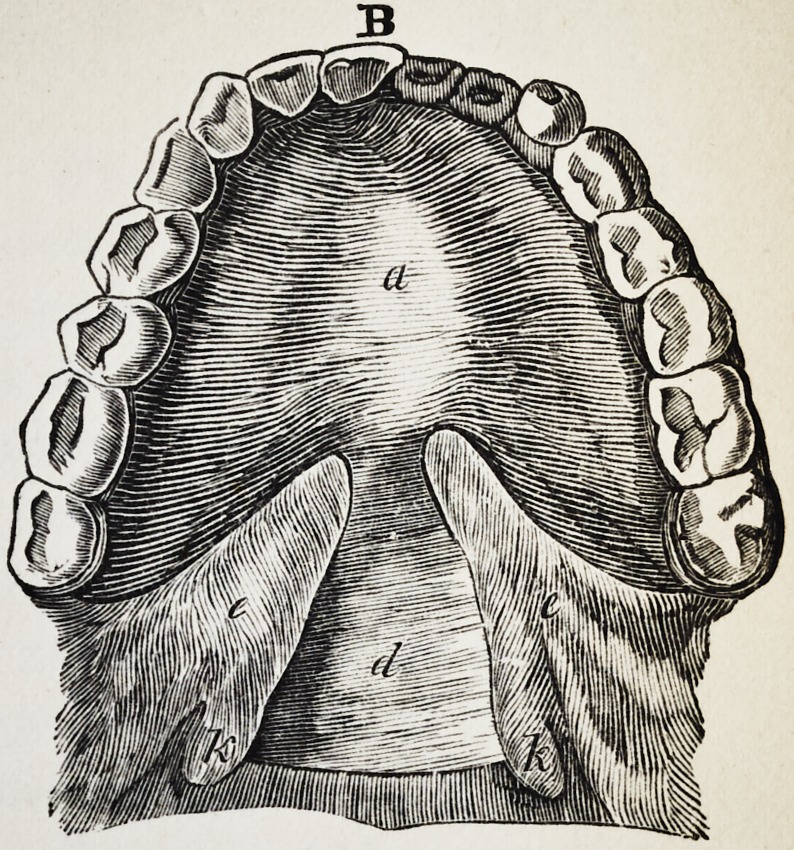


**D f10:**
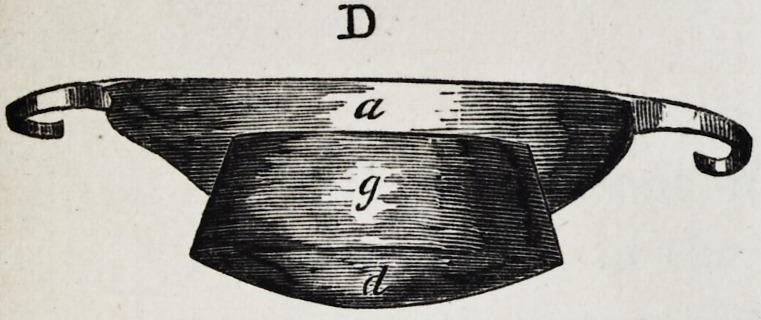


**C f11:**
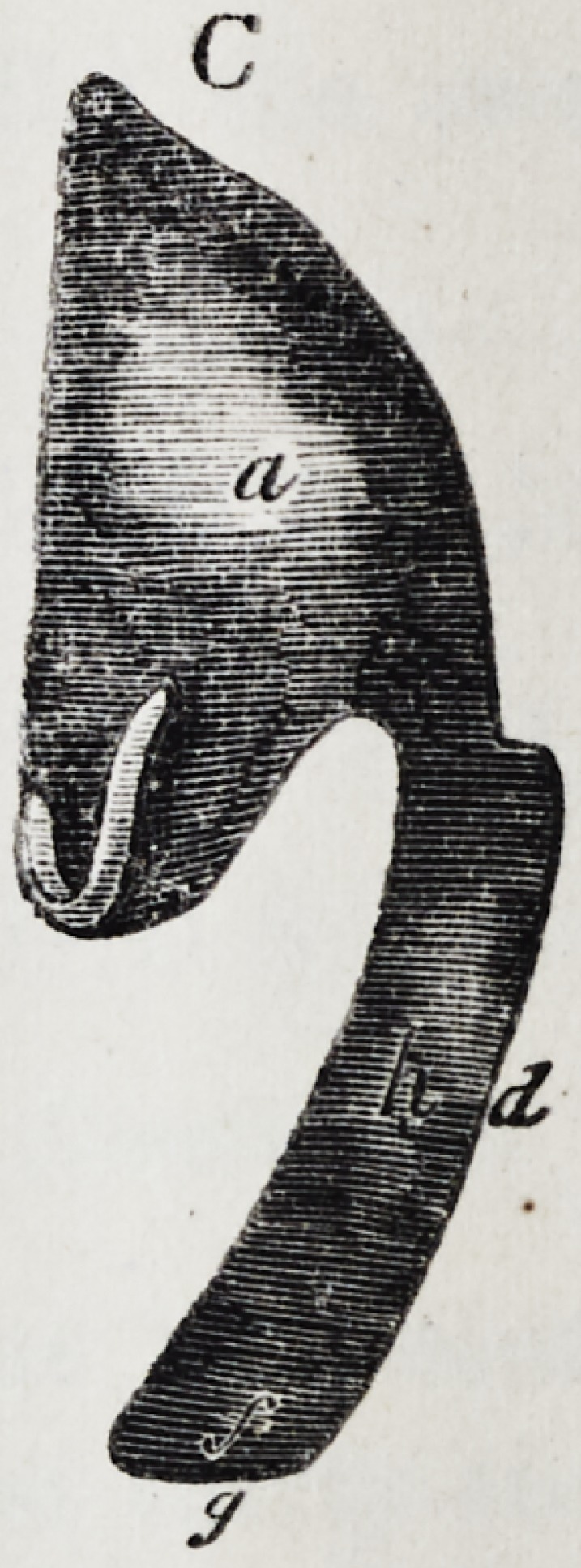


**E f12:**
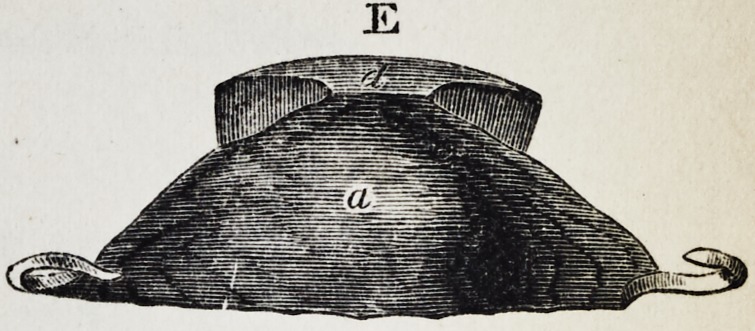


**F f13:**
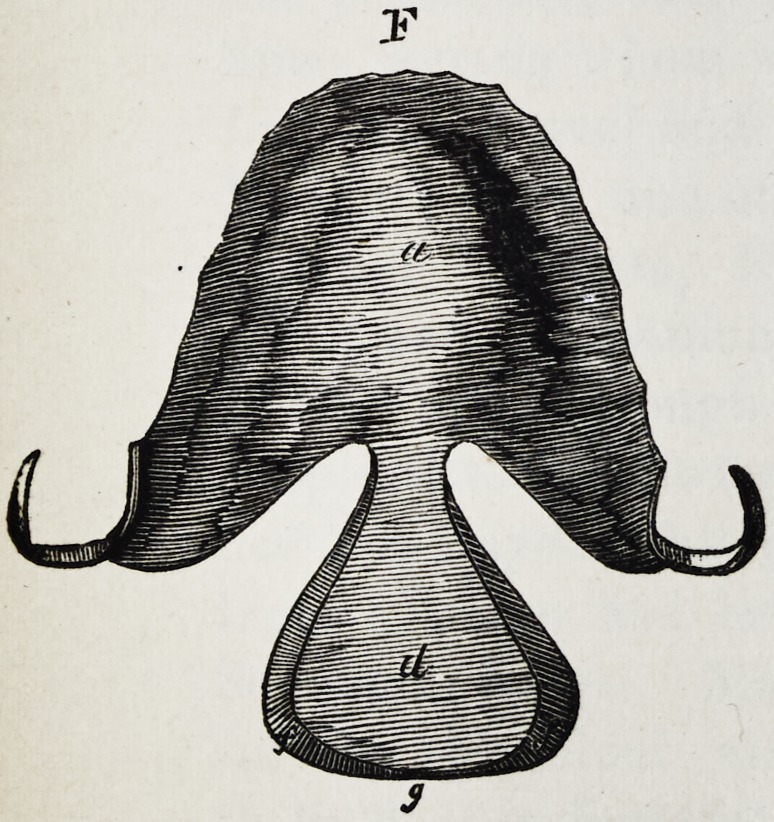


**G f14:**